# Synthesis of Alkenylgold(I) Complexes Relevant to Catalytic Carboxylative Cyclization of Unsaturated Amines and Alcohols

**DOI:** 10.3390/molecules29061331

**Published:** 2024-03-16

**Authors:** Shun Hase, Kyohei Yamashita, Yoshihito Kayaki

**Affiliations:** Department of Chemical Science and Engineering, School of Materials and Chemical Technology, Tokyo Institute of Technology, 2-12-1-E4-1 O-okayama, Meguro-ku, Tokyo 152-8552, Japan

**Keywords:** alkenylgold complex, carboxylative cyclization, carbon dioxide, cyclic urethane, cyclic carbonate, protodeauration, *N*-heterocyclic carbene ligand

## Abstract

The carboxylation of unsaturated amine and alcohol compounds, including 4-benzylamino-1-phenyl-1-butyne (homopropargylamine), 2-butyne-1-ol (propargylic alcohol), and 2,3-butadiene-1-ol (allenylmethyl alcohol), using the hydroxidogold(I) complex, AuOH(IPr) [IPr = 1,3-bis(2,6-diisopropylphenyl)-imidazol-2-ylidene], produces corresponding alkenylgold(I) complexes with a cyclic urethane or carbonate framework in high yields. The reaction takes place in aprotic THF at room temperature under the atmospheric pressure of CO_2_ in the absence of base additives. The products were characterized by NMR spectroscopy, elemental analysis, and X-ray crystallography. The functionalized alkenyl complexes prepared from the alkynes can be protonated by treatment with an equimolar amount of acetic acid to afford five- or six-membered carboxylation products, whereas the related alkenyl complex derived from allenylmethyl alcohol decomposed to recover the starting allene via ring-opening decarboxylation.

## 1. Introduction

The activation of unsaturated bonds by coordination to a Lewis acidic metal center and the following functionalization have been broadly utilized for catalytic applications. Among the intense research efforts in gold catalysis, a range of addition reactions of nucleophilic functional groups to C–C multiple bonds have been established [1,2,3,4]. In this context, organogold intermediates are proposed in mechanistic pathways involving the gold–carbon bond cleavage by a proton (i.e., protodeauration) to generate the products. Synthetic studies on alkenylgold complexes relevant to the gold-promoted transformations of alkynes and allenes have received much interest for gaining insight into catalytic behavior [5,6,7].

**Scheme 1:**
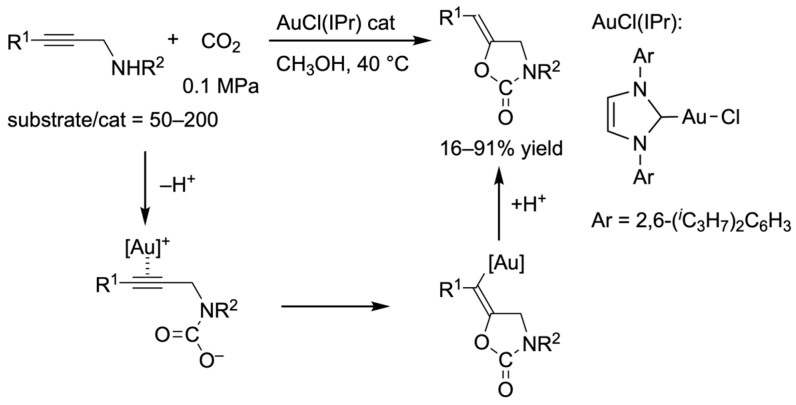
Au-catalyzed carboxylative cyclization of propargylamines.

**Scheme 2:**
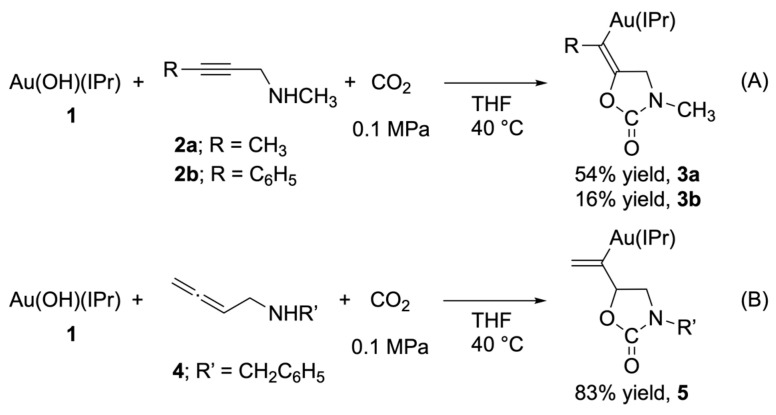
Synthesis of alkenylgold complexes using propargylic amine (**A**) and allenylmethylamine (**B**) as catalytic intermediates.

In our continuing studies on cyclic urethane synthesis from unsaturated amines and CO_2_ [8,9,10,11,12], it has been reported that gold(I) complexes bearing *N*-heterocyclic carbene (NHC) ligands serve as highly effective catalysts in alcoholic solvents for the carboxylative cyclization of propargylamines to yield 5-alkylidene-1,3-oxazolidin-2-ones with perfect regio- and stereoselectivities under mild temperature and pressure conditions (Scheme 1) [10,11]. A smooth carbamate formation from aminoalkyne and CO_2_ favors the attack of the alkyne moiety activated on the gold center, leading to the generation of alkenylgold species followed by subsequent protodeauration to release cyclic urethane. The treatment of AuOH(IPr) (**1**) and propargylic amines such as 1-(methylamino)-2-butyne (**2a**) and 1-(methylamino)-3-phenyl-2-propyne (**2b**) with atmospheric CO_2_ under aprotic and nonacidic conditions allowed the isolation of the corresponding alkenylgold(I) complexes (**3a** [10] and **3b** [11]) with a five-membered urethane moiety as the model intermediates (Scheme 2A). Separately, the related NHC–silver(I) carboxylate complexes proved to be rather effective for the carboxylative cyclization of 1-(methylamino)-2,3-butadiene (**4**; allenylmethylamine) using CO_2_ [12]. The superiority of the Ag catalyst over the Au catalyst corroborates the fact that an isolable alkenylgold complex (**5**) [12] derived from **4** and CO_2_ showed poor reactivity toward the protonolysis of the alkenyl–metal bond involved in the product-releasing step of the catalytic cycle (Scheme 2B).

These results prompted us to explore the feasibility of related alkyne or allene analogs for carbonylative cyclization using CO_2_. The identification of catalytic intermediate models via the intramolecular attack of carbamate and carbonate anions to C–C multiple bonds is an effective way to gain insight into extending the catalytic carboxylation system. In this paper, we disclose the reaction of several unsaturated amines and alcohols with a stoichiometric amount of NHC–gold(I) complex, leading to new alkenylgold complexes.

## 2. Results and Discussion

### 2.1. Synthesis and Characterization of Alkenylgold(I) Complex Derived from Homopropargylamine

**Scheme 3:**
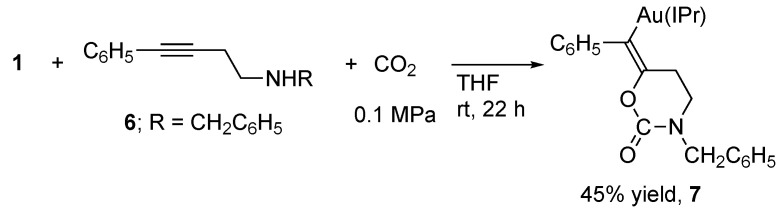
Synthesis of alkenylgold **7** from **1**, homopropargylamine, and CO_2_.

Compared to the synthesis of five-membered cyclic urethanes by the carboxylative cyclization of propargylamines, there are limited examples of the catalytic construction of six-membered variants [13,14,15,16,17,18,19]. As a model for 6-*exo*-*dig* cyclization, we initially focused on the reactivity of a one-carbon homologated aminoalkyne. Following the synthesis of alkenylgold(I) complexes from propargylic amines, we performed the reaction of homopropargylamine (**6**) with an equimolar amount of **1** under a CO_2_ atmosphere in dehydrated tetrahydrofuran (THF) at room temperature for 22 h. The desired alkenyl complex (**6**) having a six-membered urethane structure was successfully isolated as colorless and thermally stable crystals in 45% yield after recrystallization from a mixed solution of acetone and *n*-hexane (Scheme 3).

**Figure 1:**
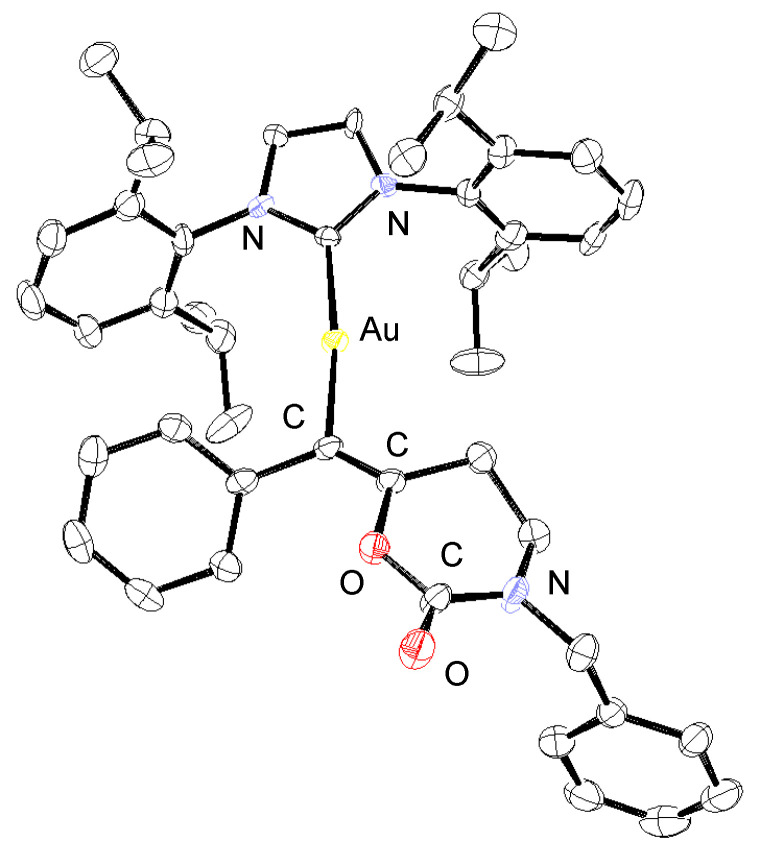
X-ray crystal structure of **7**. The hydrogen atoms are omitted for clarity. Thermal ellipsoids are shown at the 30% probability level.

The alkenylgold complex **7** was fully characterized using NMR spectroscopy, elemental analysis, and X-ray crystallography. In ^13^C{^1^H} NMR, characteristic signals at 153.1 and 195.3 ppm were ascribed to the carbene carbon bound to the Au center and the carbonyl group derived from CO_2_, respectively. The chemical shift of 127.9 ppm, attributed to the alkenyl carbon adjacent to the Au center, was reasonable relative to the signals at 131.6 and 135.9 ppm for **3a** and **3b**, respectively, with a five-membered urethane framework [11]. The composition of formal CO_2_-adduct was also confirmed by CHN elemental analysis, as well as the C=O stretching band at 1691 cm^–1^ in ATR–IR. As shown in Figure 1, the crystallographic structure of **7** revealed that the Au(I) complex adopts a two-coordinate geometry with a C–Au–C angle of 172.6(3)°. The C(alkenyl)–Au bond length of 2.056(6) Å is slightly longer than that of **3a** and **3b** (2.046 and 2.049 Å, respectively) [11], possibly due to the sterically demanding substructure of the six-membered urethane. The structural data indicate that the carboxylation of **6** provided the *anti* addition product via the nucleophilic attack of the CO_2_-derived carbamate moiety on the alkyne coordinated to the Au center.

We next examined the catalytic version of the carboxylative cyclization of homopropargylic amine. According to the original reaction of propargylamines, **6** was treated in methanol under Ar (0.1 MPa) in the presence of **1** with a substrate/catalyst ratio of 50 at 40 °C for 15 h; however, a complicated mixture was obtained. By switching the solvent to toluene, the corresponding cyclic urethane was obtained as the 6-*exo*-dig cyclization product in 28% yield and 22% of the unreacted substrate still remained in the reaction mixture. Due to a low catalytic activity of the Au complex, the carboxylation could not be completed even under pressurized CO_2_ (3.0 MPa).

### 2.2. Synthesis and Characterization of Alkenylgold(I) Complex Derived from Propargy Alcohol

**Scheme 4:**
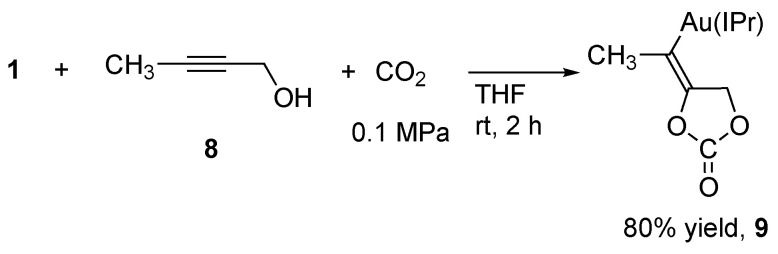
Synthesis of alkenylgold **9** from **1**, propargylic alcohol, and CO_2_.

The carboxylative cyclization of propargylic alcohols has also been investigated using metal and base catalysts. In most catalytic systems, tertiary alcohols have been exclusively used as the substrates, whereas few examples have been reported for the synthesis of cyclic carbonate from primary propargylic alcohol [20,21,22,23]. In light of the potential for expanding the substrate scope, we next explore the synthesis of alkenylgold from CO_2_ and 2-butyne-1-ol (**8**) without the virtue of the Thorpe–Ingold effect. When stoichiometric carboxylative cyclization was performed by the treatment of **1** with **8** in THF under a CO_2_ atmosphere for 1 h, the desired alkenylgold complex (**9**) was formed and successfully isolated in 80% yield after recrystallization (Scheme 4). The ^13^C{^1^H} NMR spectrum in CDCl_3_ displayed a carbonate resonance at 155.7 ppm, along with a signal at 195.8 ppm ascribed to the carbene carbon bound to the Au center. Two alkenyl carbon signals observed at 131.7 and 139.0 ppm were identical to those at 131.6 and 139.6 ppm for the carbamate analog **3a** derived from 1-(methylamino)-2-butyne.

**Figure 2:**
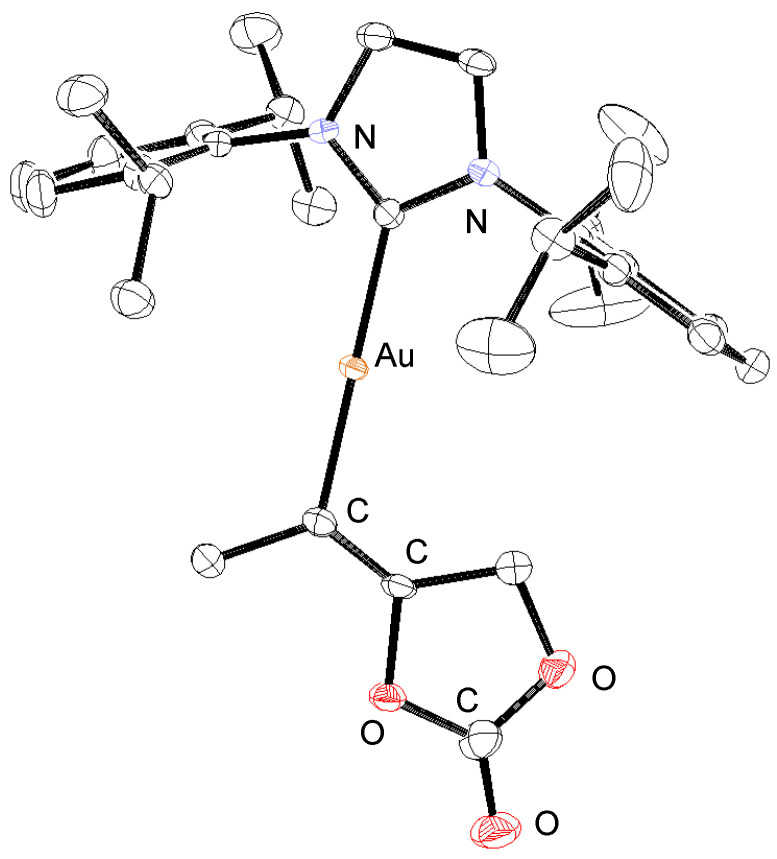
X-ray crystal structure of **9**. The hydrogen atoms are omitted for clarity. Thermal ellipsoids are shown at the 30% probability level.

A single-crystal X-ray diffraction analysis revealed that the structure of **9** resembles that of the monomeric alkenylgold complex of **3a**, as shown in Figure 2. The bond lengths and angles within the alkenyl ligand (Table S2) were roughly similar to those of the urethane analog **3a** [11]. The sum of angles around each alkene carbon atom is consistent with a planar geometry of the alkenyl ligand. A comparable distance (1.432(5) Å) between the alkenyl carbon atom at the β-position to the Au center and the carbonate oxygen atom originated from CO_2_ was identified, relative to the corresponding bond length of 1.437 Å (mean value) in the carbamate derivative of **3a** [11]. These results are indicative of the smooth capture of CO_2_ at the alcoholic moiety followed by nucleophilic cyclization to construct the five-membered carbonate ring.

In the trial catalytic carbonate synthesis from **8** and CO_2_ (5.0 MPa) with reference to the carboxylative cyclization of propargylic amines [10], the alcoholic substrate was mostly recovered from the reaction mixture containing a catalytic amount of **9** in methanol even after an elongated reaction time of 48 h. The alcohol, unlike unsaturated amines, was less susceptible to the catalytic release of cyclic carbonate, while the basicity of hydroxidogold complex possibly contributes to enhancing the nucleophilicity of propargylic alcohols for carboxylation.

### 2.3. Synthesis and Characterization of Alkenylgold(I) Complex Derived from Allenylmethyl Alcohol

**Scheme 5:**
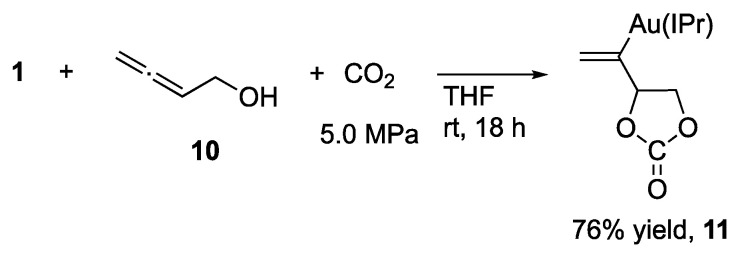
Synthesis of alkenylgold **11** from **1**, allenylmethyl alcohol, and CO_2_.

The carboxylative cyclization of allenylated alcohols has not been reported (related cyclic carbonate synthesis from allenyl alcohols and CO_2_ via Mizoroki-Heck type reaction was reported by Inoue, et al., as cited in ref. [24]), whereas allenylmethylamine has successfully been converted into corresponding cyclic urethane. To investigate nucleophilic attack on the carbon–carbon double bond by a carbonate moiety generated from CO_2_, we next focused on the reaction of allenylmethyl alcohol (**10**). In a similar manner to the synthesis of **7** and **9**, the reaction of **1** and **10** proceeded in THF under CO_2_ (5.0 MPa) at room temperature to give a colorless solution. After the evaporation of the solvent, the desired alkenylgold complex (**11**) having a cyclic carbonate structure was isolated in 76% yield as crystals which were formed by slow diffusion of *n*-pentane into the solution in acetone (Scheme 5).

**Figure 3:**
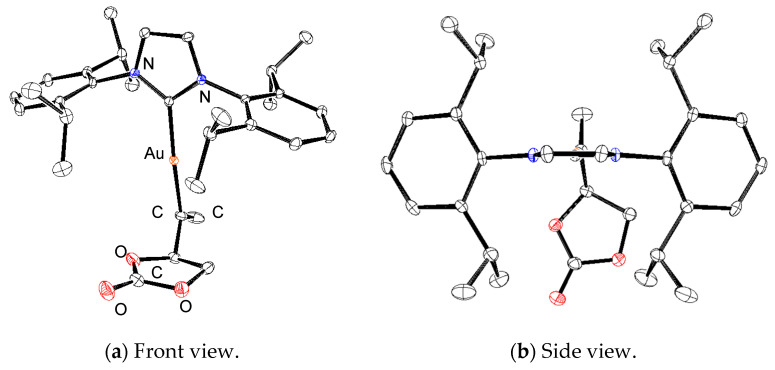
X-ray crystal structure of **11**. The hydrogen atoms are omitted for clarity. Thermal ellipsoids are shown at the 30% probability level.

The product was characterized by NMR spectroscopy, elemental analysis, and X-ray crystallography. In the ^1^H NMR spectrum of **11** in CDCl_3_, the unsymmetrical terminal vinyl protons appeared as double doublet signals at 4.61 and 5.47 ppm with ^2^*J*_HH_ and ^4^*J*_HH_ couplings, which is comparable to those at 4.46 and 5.35 ppm for the alkenylgold complex **5** [12] synthesized from allenylmethylamine. Compared to the alkenylgold isomer **9**, a marked downfield shift of the ^13^C{^1^H} NMR signal to 172.0 ppm, which was assigned to the alkenyl carbon adjacent to the Au center, was observed, as with the case of **5** (175.4 ppm) having a cyclic urethane framework. The carbonate signal appeared at 156.1 ppm in a similar manner to that of **9** (155.7 ppm). In addition, the carbonate function was confirmed by a C=O stretching frequency at 1782 cm^–1^ in ATR–IR. As shown in Figure 3a, the X-ray crystallographic structure shows a typical two-coordinate geometry around the Au center attached to the NHC carbene and alkenyl carbons. The distance between alkene carbons (1.322(6) Å) is close to that of other alkenyl complexes including the isomer **9** (1.321(5) Å). Other bond lengths and angles within the alkenyl ligand (Table S3) were roughly similar to those of the urethane analog **5** [12]. Apart from the alkenyl complexes (**3a**, **3b**, **5**, **7**, and **9**) displaying similar structures where the urethane or carbonate ring and the NHC ligand lie in the nearly same plane, the carbonate ring and double bond of the alkenyl ligand in **11** are perpendicular to the NHC ring, as illustrated in Figure 3b.

### 2.4. Protonolysis of Alkenylgold Complexes ***7***, ***9***, and ***11***

**Scheme 6:**
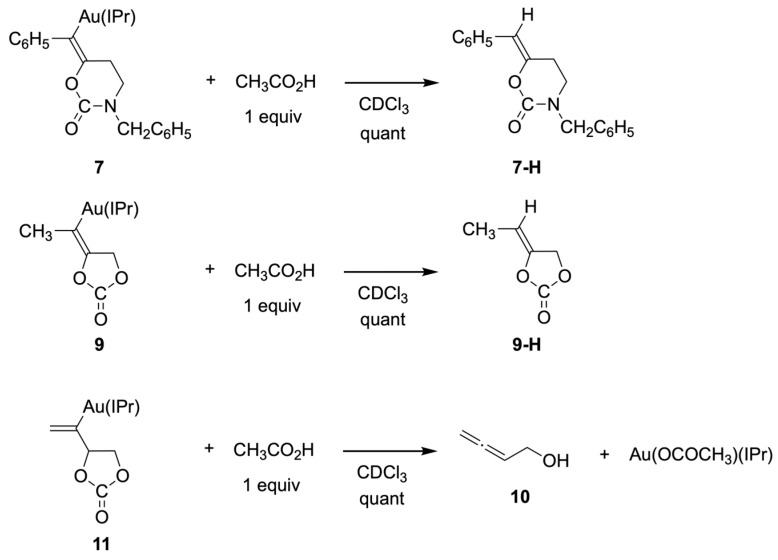
Protonolysis of alkenylgold complexes **7**, **9**, and **11**.

Alkenylgold complexes produce corresponding alkenes via the protodeauration step in catalysis [25,26,27]. For example, the complexes **2a** and **2b** produced corresponding urethanes via protonolysis and showed catalytic reactivity in the carboxylative cyclization of propargylamines. Therefore, we tested the protonolysis of the obtained alkenylgold complexes as the models of the new catalytic carboxylative cyclizations. NMR monitoring experiments by the treatment of **7** or **9** with an equimolar amount of acetic acid (p*K*a = 4.76 in H_2_O) in CDCl_3_ showed that the protodeauration proceeded to yield only *Z* products (**7-H** and **9-H**) quantitatively (Scheme 6). Contrastingly, the reaction of **11** with acetic acid produced the parent allenyl alcohol **10** and Au(OCOCH_3_)(IPr) readily and quantitatively via ring opening and decarboxylation, owing to the relatively weak C–O bond in the allylic carbonate.

## 3. Materials and Methods

All syntheses were performed under a purified argon atmosphere using standard Schlenk techniques. 2-Butyn-1-ol (**8**) was purchased from Tokyo Chemical Industry Co., Ltd. (Tokyo, Japan), and dried by calcium hydride and distilled under argon. 1-Benzylamino-4-phenyl-3-butyne (**6**) [28] and 1-hydroxy-2,3-butadiene (**10**) [29] were prepared according to the literature. Solvents were purchased from Kanto Chemical Co., Inc. (Tokyo, Japan) and dried by refluxing over sodium benzophenone ketyl (THF, diethyl ether, and toluene), acetone (B_2_O_3_), or CaH_2_ (methanol, hexane, and pentane) and distilled under argon before use. Carbon dioxide (99.999%) was purchased from Resonac Gas Products Corp. (Kawasaki, Japan). Au(OH)(IPr) (**1**) [30] was prepared according to the literature. ^1^H (399.8 MHz) and ^13^C{^1^H} (100.5 MHz) NMR spectra were acquired on a JNM-ECX400 spectrometer (JEOL Ltd., Tokyo, Japan) as solutions in CDCl_3_. The NMR chemical shifts were referenced to an external tetramethylsilane signal (0.0 ppm) by using the signals of residual proton impurities in the deuterated solvents. Elemental analyses were carried out using a PE2400 Series II CHNS/O Analyzer (PerkinElmer, Inc., Waltham, MA, USA). IR spectra were recorded on a JASCO FT/IR-610 spectrometer (JASCO Corporation, Tokyo, Japan).

### 3.1. Synthesis of the Alkenylgold Complexes

**Complex 7**: 1-Benzylamino-4-phenyl-3-butyne **6** (120 mg, 0.51 mmol) was added to a THF (5 mL) solution of **1** (312 mg, 0.52 mmol) in a 20 mL Schlenk flask under an Ar atmosphere. The Schlenk flask was charged with CO_2_ and stirred at room temperature for 22 h. The resulting mixture was evaporated and washed with hexane with a small portion of ether. Colorless crystals of **7** were collected by crystallization from acetone/hexane (200 mg, 45% yield). ^1^H NMR (399.78 MHz, CDCl_3_, rt): *δ* 1.20 (d, ^3^*J*_HH_ = 6.9 Hz, 12H, (C*H*_3_)_2_CH), 1.25 (d, ^3^*J*_HH_ = 6.9 Hz, 12H, (C*H*_3_)_2_CH), 2.21 (t, ^3^*J*_HH_ = 6.1 Hz, 2H, COC*H*_2_CH_2_), 2.59 (sept, ^3^*J*_HH_ = 6.9 Hz, 4H, (CH_3_)_2_C*H*), 2.83 (t, ^3^*J*_HH_ = 6.1 Hz, 2H, CH_2_C*H*_2_N), 4.48 (s, 2H, NC*H*_2_C_6_H_5_), 6.84 (t, ^3^*J*_HH_ = 7.3 Hz, 1H, Ar), 6.93 (t, *J*_HH_ = 7.5 Hz, 2H, Ar), 7.03 (dd, *J*_HH_ = 8.2 Hz, 1.2 Hz, 2H, Ar), 7.16 (s, 2H, NC*H*=C*H*N), 7.24-7.34 (m, 7H, Ar), 7.46 (t, ^3^*J*_HH_ = 7.8 Hz, 2H, Ar); ^13^C{^1^H} NMR (100.53 MHz, CDCl_3_, rt): *δ* 24.1, 24.2, 28.8, 30.1, 44.4, 52.4, 58.3, 122.6, 123.5, 123.9, 126.8, 127.2, 127.9 (Au*C*=C), 128.5, 130.0, 130.1, 134.0, 134.6, 137.3, 142.8, 143.5 (AuC=*C*), 144.8, 145.9, 153.1 (*C*=O), 195.3 (N*C*N). IR (cm^−1^, KBr): 1691 (C=O). Anal. Calcd for C_45_H_52_AuN_3_O_2_ (863.90): C, 62.56; H, 6.07; N, 4.86. Found: C, 62.35; H, 6.34; N, 4.84.

**Complex 9**: 2-Butyne-1-ol (40 mg, 0.57 mmol) was added to a THF (3 mL) solution of **1** (304 mg, 0.50 mmol) in a 20 mL Schlenk flask under Ar atmosphere. The Schlenk flask was charged with CO_2_ and stirred at rt for 2 h. The resulting mixture was evaporated and washed with ether. Colorless crystals of **9** were collected by crystallization from wet acetone/hexane (281 mg, 80% yield). ^1^H NMR (399.78 MHz, CDCl_3_, rt): *δ* 1.23 (d, ^3^*J*_HH_ = 6.9 Hz, 12H, (C*H*_3_)_2_CH), 1.32 (d, ^3^*J*_HH_ = 6.9 Hz, 12H, (C*H*_3_)_2_CH), 1.56 (t, 3H, ^5^*J*_HH_ = 2.0 Hz, C*H*_3_C=C), 2.64 (sept, ^3^*J*_HH_ = 6.9 Hz, 4H, (C*H*_3_)_2_CH), 4.16 (q, ^5^*J*_HH_ = 2.0 Hz, 2H, C*H*_2_), 7.15 (s, 2H, NC*H*=C*H*N), 7.30 (d, ^3^*J*_HH_ = 7.7 Hz, 4H, Ar), 7.51 (t, ^3^*J*_HH_ = 7.7 Hz, 2H, Ar); ^13^C{^1^H} NMR (100.53 MHz, CDCl_3_, rt): *δ* 19.1, 24.1, 24.5, 28.9, 68.1, 122.9, 124.0, 130.5, 131.7 (Au*C*=C), 134.4, 139.0 (AuC=*C*), 145.9, 155.7 (*C*=O), 195.8(N*C*N). IR (cm^−1^, KBr): 1799 (C=O). Anal. Calcd for C_32_H_41_AuN_2_O_3_+H_2_O (716.67): C, 53.63; H, 6.05; N, 3.91. Found: C, 53.55; H, 5.88; N, 3.86.

**Complex 11**: 1-Hydroxy-2,3-butadiene **10** (14 mg, 0.20 mmol) was added to a THF (1 mL) solution of **1** (103 mg, 0.17 mmol) in a 50 mL steel autoclave under Ar atmosphere. The reaction mixture was charged with pressurized CO_2_ (5.0 MPa) and stirred at rt for 18 h. The resulting mixture was evaporated and washed with hexane. Colorless crystals of **11** were collected by crystallization from acetone/pentane (91 mg, 76%). Colorless crystals. ^1^H NMR (399.78 MHz, CD_2_Cl_2_, rt): *δ* 1.22 (d, ^3^*J*_HH_ = 7.0 Hz, 12H; (C*H*_3_)_2_CH), 1.33 (d, ^3^*J*_HH_ = 6.7 Hz, 12H; (C*H*_3_)_2_CH), 2.57 (overlapped sept, ^3^*J*_HH_ = 6.7 Hz, 4H; (CH_3_)_2_C*H*), 3.44 (dd, ^3^*J*_HH_ = 8.3 Hz, ^3^*J*_HH_ = 7.9 Hz, 1H; COC*H*H), 3.88 (dd, ^3^*J*_HH_ = 8.1 Hz, ^3^*J*_HH_ = 8.1 Hz, 1H; COCH*H*), 4.61 (dd, ^2^*J*_HH_ = 4.1 Hz, ^4^*J*_HH_ = 1.4 Hz, 1H; C*H*H=CAu), 4.86 (dd, ^3^*J*_HH_ = 8.2 Hz, ^3^*J*_HH_ = 8.2 Hz, 1H; CH_2_=CC*H*), 5.47 (dd, ^2^*J*_HH_ = 4.0 Hz, ^4^*J*_HH_ = 1.3 Hz, 1H; CH*H*=CAu), 7.16 (s, 2H; NC*H*=C*H*N), 7.29 (d, ^3^*J*_HH_ = 7.8 Hz. 4H; Ar), 7.51 (t, ^3^*J*_HH_ = 7.8 Hz, 2H; Ar) ppm. ^13^C{^1^H} NMR (100.53 MHz, CD_2_Cl_2_, rt): *δ* 24.0, 24.4, 28.73, 28.77, 70.4 (CH*C*H_2_O), 87.0 (AuC*C*HO), 120.8, 122.9, 123.9, 124.0, 134.2, 145.69, 145.72, 156.0 (O*C*O_2_), 172.0 (Au*C*=CH_2_), 195.8 (N*C*N) ppm. IR (cm^−1^, ATR): 1782 (C=O). Anal. Calcd for C_32_H_41_AuN_2_O_3_ (698.66): C, 55.01; H, 5.92; N, 4.01. Found: C, 55.21; H, 6.14; N, 4.00.

### 3.2. Protonolysis of the Alkenylgold Complexes with Acetic Acid

An NMR tube equipped with a J-Young valve was charged with alkenylgold complex (10 μmol), durene and CDCl_3_ (0.5 mL), and acetic acid (10 μmol) was added to the solution. The reaction was monitored via ^1^H NMR spectroscopy at room temperature.

**7-H:** ^1^H NMR (399.78 MHz, CDCl_3_, rt): *δ* 2.66 (t, ^3^*J*_HH_ = 6.1 Hz, 2H; COC*H*_2_CH_2_), 3.29 (t, ^3^*J*_HH_ = 6.1 Hz, 2H; CH_2_C*H*_2_N), 4.63 (s, 2H; NC*H*_2_C_6_H_5_), 5.43 (s, 1H; C_6_H_5_C*H*), 7.19-7.23 (m, 1H; Ar), 7.26-7.30 (m, 7H; Ar), 7.66 (dd, *J*_HH_ = 8.7 Hz, *J*_HH_ = 1.3 Hz, 1H; Ar).

**9-H:** ^1^H NMR (399.78 MHz, CDCl_3_, rt): *δ* 1.73 (dt, ^3^*J*_HH_ = 7.0 Hz, ^5^*J*_HH_ = 2.1 Hz, 3H; C*H*_3_), 4.72 (qt, ^3^*J*_HH_ = 7.0 Hz, ^4^*J*_HH_ = 2.1 Hz, 1H; CH_3_C*H*), 4.95 (dq, ^4^*J*_HH_ = 2.1 Hz, ^5^*J*_HH_ = 2.1 Hz, 2H; C*H*_2_O).

### 3.3. X-ray Crystal Structure Determination

Diffraction experiments were performed on a Rigaku Saturn CCD area detector (Rigaku Corporation, Tokyo, Japan) using graphite-monochromated Mo-*K*α radiation (λ = 0.71075 Å) under a nitrogen stream at 193 K. Single crystals suitable for X-ray analyses were mounted on glass fibers. The crystal-to-detector distance was 45.0 mm. Data were collected to a maximum 2*θ* value of 55.0°. A total of 720 oscillation images were collected. A sweep of the data was carried out by using ω scans from −110.0 to 70.0° in 0.5° steps at *χ* = 45.0° and *ϕ* = 0.0°. A second sweep was performed by using ω scans from −110.0 to 70.0° in 0.5° steps at *χ* = 45.0° and *ϕ* = 90.0°. Intensity data were collected for Lorentz-polarization effects and absorption. Details of the crystal and data collection parameters for the compounds **7**, **9**, and **11** are summarized in Table S1. The structure solution and refinements were performed with the CrystalStructure program package [31]. The heavy atom positions were determined by a direct program method (SIR92) [32] and the remaining non-hydrogen atoms were found by subsequent Fourier syntheses and were refined by full-matrix least-squares techniques against *F*^2^ using the SHELXL-2014/7 program [33]. The hydrogen atoms were placed at calculated positions and were refined with a riding model. These crystallographic data have been deposited with Cambridge Crystallographic Data Centre as supplementary publication numbers CCDC-2339858 (**7**), CCDC-2339859 (**9**), and CCDC-2339860 (**11**).

## 4. Conclusions

Functionalized alkenylgold(I) complexes can be versatilely synthesized by the reaction of AuOH(IPr) and unsaturated amines or alcohols under a CO_2_ atmosphere. The isolated complexes are regarded as possible catalytic intermediates for the carboxylative cyclization using CO_2_. The feasibility of this method for Au catalysis can be evaluated by performing protonolysis, which is the fundamental process in catalytic carboxylative cyclization. The carboxylative cyclizations of homopropargylamine and primary propargylic alcohol were demonstrated by stepwise stoichiometric carboxyauration and protonolysis. Based on these fundamental aspects on the catalytic carboxylative cyclizations, further investigations involving the transformation of the related alkynes and alkenes are now underway.

## Data Availability

The data presented in this study are available in the Supplementary Materials.

## Supplementary Materials

The following supporting information can be downloaded at: https://www.mdpi.com/article/10.3390/molecules29061331/s1, Table S1: X-ray crystallographic data table for **7**, **9**, and **11**; Figures S1–S3: Selected bond lengths and angles for **7**, **9**, and **11**. File S1–S3: checkcif.
