# Supplementary material for: Synthesis of Alkenylgold(I) Complexes Relevant to Catalytic Carboxylative Cyclization of Unsaturated Amines and Alcohols
Source: Molecules. 2024 Mar 16;29(6):1331. doi: 10.3390/molecules29061331 (PMC10975222; doi:10.3390/molecules29061331)

checkCIF/PLATON report

You have not supplied any structure factors. As a result the full set of tests cannot be run.

THIS REPORT IS FOR GUIDANCE ONLY. IF USED AS PART OF A REVIEW PROCEDURE FOR PUBLICATION, IT SHOULD NOT REPLACE THE EXPERTISE OF AN EXPERIENCED CRYSTALLOGRAPHIC REFEREE.

No syntax errors found. CIF dictionary Interpreting this report

Datablock: Au-6membered-carbamatev7

Bond precision: C-C = 0.0105 A Wavelength=0.71075

Cell:	a=12.504(2)	b=19.815(4)	c=16.172(3)
	alpha=90	beta=97.681(2)	gamma=90
Temperature:	93 K		

	Calculated	Reported
Volume	3970.9(13)	3970.8(12)
Space group	P 21/n	P 1 21/n 1
Hall group	-P 2yn	-P 2yn
Moiety formula	C45 H52 Au N3 O2	C45 H52 Au N3 O2
Sum formula	C45 H52 Au N3 O2	C45 H52 Au N3 O2
Mr	863.87	863.89
Dx,g cm-3	1.445	1.445
Z	4	4
Mu (mm-1)	3.745	3.758
F000	1752.0	1752.0
F000'	1744.67	
h,k,lmax	16,25,20	16,25,20
Nref	9088	8978
Tmin,Tmax	0.668,0.769	0.632,0.769
Tmin'	0.655	

Correction method= # Reported T Limits: Tmin=0.632 Tmax=0.769
AbsCorr = MULTI-SCAN

Data completeness= 0.988 Theta(max)= 27.470

R(reflections)= 0.0581(7443) wR2(reflections)= 0.1345(8978)

S = 1.000 Npar= 512

The following ALERTS were generated. Each ALERT has the format
test-name_ALERT_alert-type_alert-level.
Click on the hyperlinks for more details of the test.

Alert level C

```
PLAT342_ALERT_3_C Low Bond Precision on  C-C Bonds ...............    0.01055 Ang.
```

Alert level G

```
CHEMS02_ALERT_1_G  Please check that you have entered the correct
            _publ_requested_category classification of your compound;
            FI or CI or EI for inorganic; FM or CM or EM for metal-organic;
            FO or CO or EO for organic.
            From the CIF: _publ_requested_category   CHOOSE FI FM FO CI CM CO or A
            From the CIF: _chemical_formula_sum :C45 H52 Au1 N3 O2
PLAT005_ALERT_5_G No Embedded Refinement Details Found  in the CIF     Please Do !
PLAT882_ALERT_1_G No Datum for _diffrn_reflns_av_unetI/netI ......     Please Do !
PLAT963_ALERT_2_G Both SHELXL WEIGHT Parameter Values Zero .......     Please Check
```

0 **ALERT level A** = Most likely a serious problem - resolve or explain
0 **ALERT level B** = A potentially serious problem, consider carefully
1 **ALERT level C** = Check. Ensure it is not caused by an omission or oversight
4 **ALERT level G** = General information/check it is not something unexpected

2 ALERT type 1 CIF construction/syntax error, inconsistent or missing data
1 ALERT type 2 Indicator that the structure model may be wrong or deficient
1 ALERT type 3 Indicator that the structure quality may be low
0 ALERT type 4 Improvement, methodology, query or suggestion
1 ALERT type 5 Informative message, check

It is advisable to attempt to resolve as many as possible of the alerts in all categories. Often the minor alerts point to easily fixed oversights, errors and omissions in your CIF or refinement strategy, so attention to these fine details can be worthwhile. In order to resolve some of the more serious problems it may be necessary to carry out additional measurements or structure refinements. However, the purpose of your study may justify the reported deviations and the more serious of these should normally be commented upon in the discussion or experimental section of a paper or in the "special_details" fields of the CIF. checkCIF was carefully designed to identify outliers and unusual parameters, but every test has its limitations and alerts that are not important in a particular case may appear. Conversely, the absence of alerts does not guarantee there are no aspects of the results needing attention. It is up to the individual to critically assess their own results and, if necessary, seek expert advice.

Publication of your CIF in IUCr journals

A basic structural check has been run on your CIF. These basic checks will be run on all CIFs submitted for publication in IUCr journals (*Acta Crystallographica*, *Journal of Applied Crystallography*, *Journal of Synchrotron Radiation*); however, if you intend to submit to *Acta Crystallographica Section C* or *E* or *IUCrData*, you should make sure that full publication checks are run on the final version of your CIF prior to submission.

Publication of your CIF in other journals

Please refer to the *Notes for Authors* of the relevant journal for any special instructions relating to CIF submission.

PLATON version of 06/01/2024; check.def file version of 05/01/2024

Datablock Au-6membered-carbamatev7 - ellipsoid plot

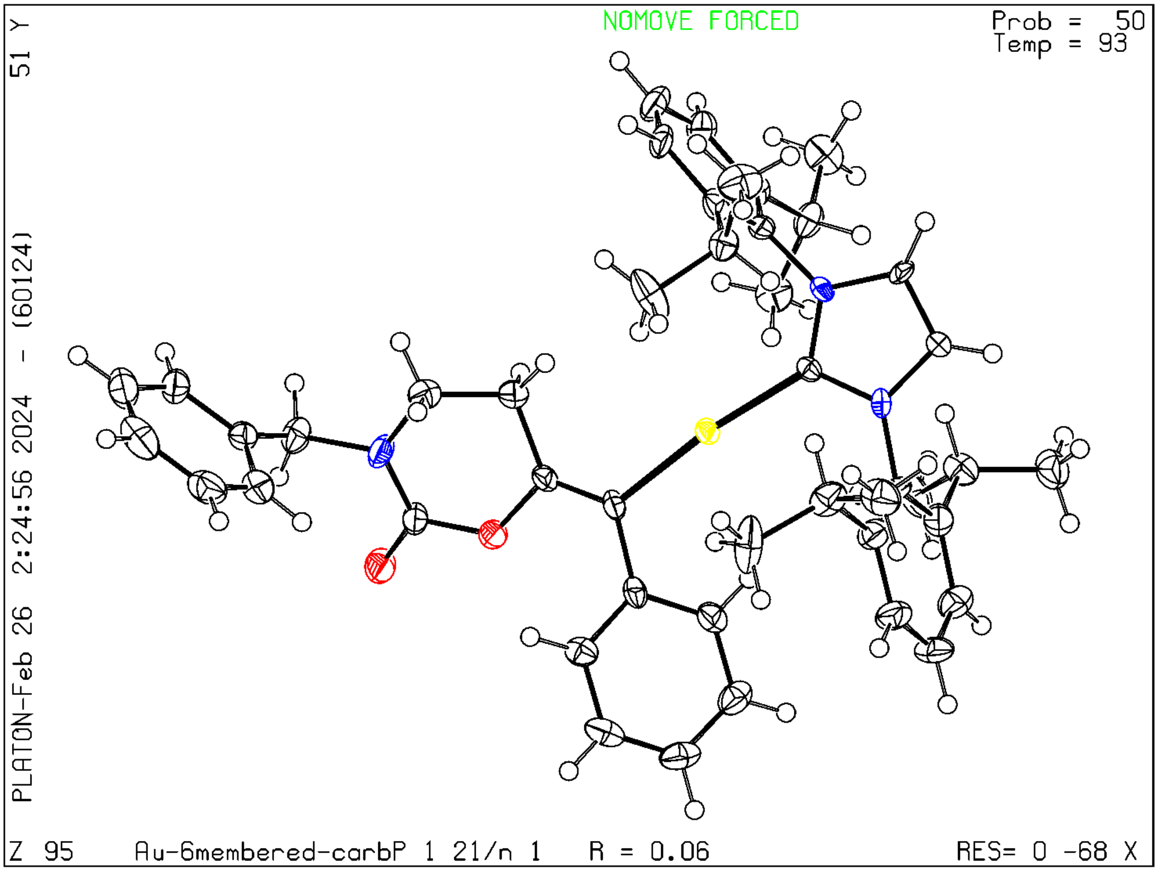

Supplement: Supplementary file 1 [file molecules-29-01331-s001.zip › File S1-7-checkcif.pdf]
