# Supplementary material for: Synthesis of Alkenylgold(I) Complexes Relevant to Catalytic Carboxylative Cyclization of Unsaturated Amines and Alcohols
Source: Molecules. 2024 Mar 16;29(6):1331. doi: 10.3390/molecules29061331 (PMC10975222; doi:10.3390/molecules29061331)
Supplement: Supplementary file 1 [file molecules-29-01331-s001.zip › molecules-2913977-Supplementary Materials.pdf]

Supplementary Materials

Synthesis of Alkenylgold(I) Complexes Relevant to Catalytic Carboxylative Cyclization of Unsaturated Amines and Alcohols

Shun Hase, Kyohei Yamashita, and Yoshihito Kayaki*

Department of Chemical Science and Engineering, School of Materials and Chemical Technology, Tokyo Institute of Technology, 2-12-1-E4-1 O-okayama, Meguro-ku Tokyo 152-8552, Japan
*E-mail: ykayaki@o.cc.titech.ac.jp

Contents

	Page
X-ray Crystallographic Data	S2

X-ray Crystallographic Data

Table S1. Crystallographic data for **7**, **9**, and **11**

	**7**	**9**	**11**
Empirical Formula	$C_{45}H_{52}AuN_3O_2$	$C_{32}H_{41}AuN_2O_3$	$C_{32}H_{41}AuN_2O_3$
Formula Weight	863.89	698.65	698.65
Crystal Color, Habit	colorless, prism	colorless, prism	colorless, prism
Crystal System	monoclinic	orthorhombic	monoclinic
Space Group	$P2_1/n$ (#14)	*P*bca (#61)	$P2_1/n$ (#14)
Lattice Parameters	a = 12.504(2) Å	a = 15.729(2) Å	a = 10.153(3) Å
	b = 19.815(4) Å	b = 17.415 (2) Å	b = 16.657(4) Å
	c = 16.172(3) Å	c = 22.599(3) Å	c = 18.566(4) Å
	β = 97.681(2) °		β = 95.620(4) °
	V = 3970.8(12) Å^3	V = 6190.7(10) Å^3	V = 3165.0(13) Å^3
Z value	4	8	4
D_{calc}	1.445 g/cm^3	1.499 g/cm^3	1.485 g/cm^3
F_{000}	1752.00	2800.00	1400.00
μ(Mo*K*α)	37.576 cm^{-1}	48.019 cm^{-1}	47.563 cm^{-1}
Exposure Rate	10.0 sec./°	16.0 sec./°	16.0 sec./°
No. of Reflections Measured	32619	48693	25391
No. of Unique Reflections	8978	7068	7030
No. Variables	512	384	343
$R1$ ($I>2.00\sigma(I)$)	0.0581	0.0310	0.0484
w$R2$ (All Reflections)	0.1345	0.0897	0.1322
Max Shift/Error in Final Cycle	0.000	0.000	0.002
GOF on F^2	1.000	1.000	0.894

$R1 = \Sigma ||F_o| - |F_c|| / \Sigma |F_o|$, $wR2 = [\Sigma (w (F_o^2 - F_c^2)^2)/ \Sigma w(F_o^2)^2]^{1/2}$.

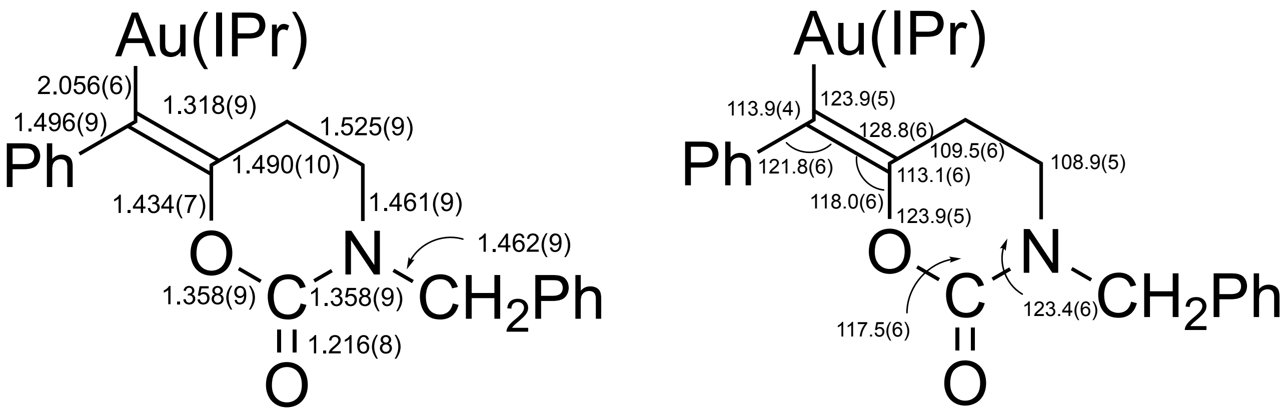

Figure S1. Selected bond lengths (Å; left) and angles (°; right) for **7**.

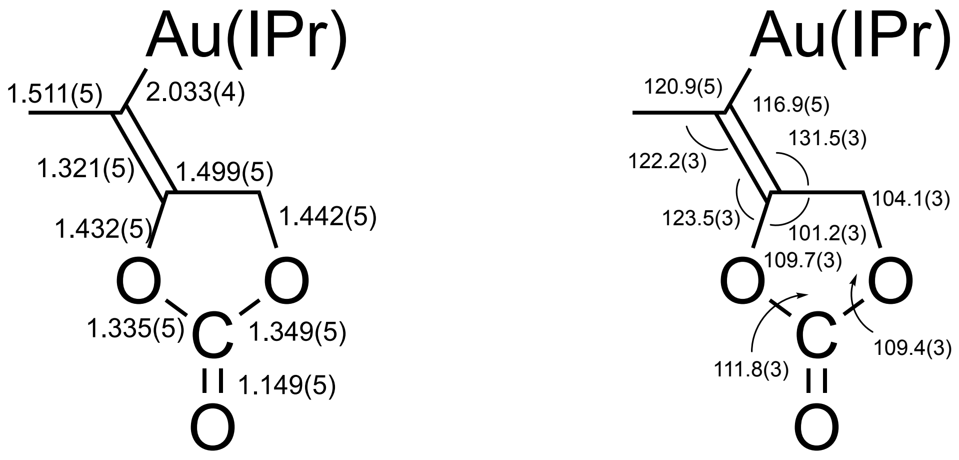

Figure S2. Selected bond lengths (Å; left) and angles (°; right) for **9**.

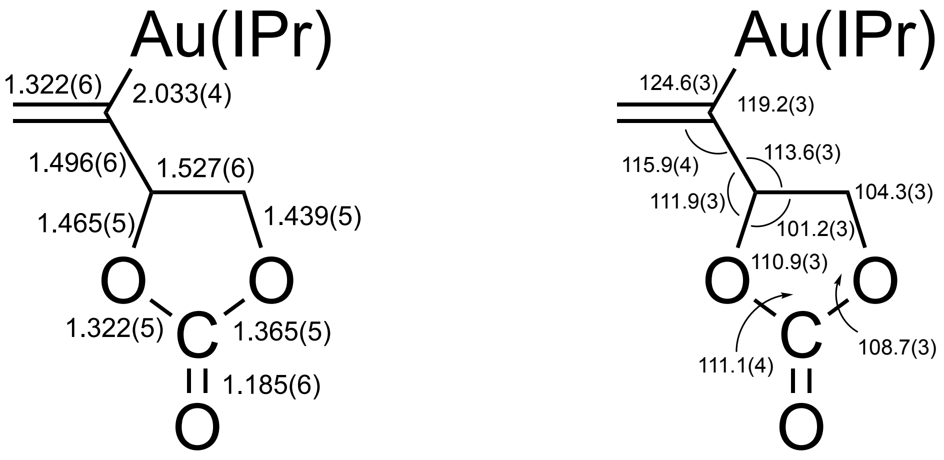

Figure S3. Selected bond lengths (Å) and angles (°) for **11**.
